# Low Performance of a Clinical-Genetic Model in the Estimation of Time in Therapeutic Range in Acenocoumarol-Adherent Patients with Nonvalvular Atrial Fibrillation: The Quality of Anticoagulation Challenge

**DOI:** 10.1155/2018/8012747

**Published:** 2018-10-17

**Authors:** Samantha Wasniewski, Luciano Consuegra-Sánchez, Pablo Conesa-Zamora, Luis García de Guadiana-Romualdo, Pablo Ramos-Ruiz, Marta Merelo-Nicolás, F. Guillermo Clavel-Ruipérez, Begoña Alburquerque-González, Federico Soria-Arcos, Juan A. Castillo-Moreno

**Affiliations:** ^1^Department of Cardiology, Santa Lucía General University Hospital, Cartagena-Murcia, Spain; ^2^Department of Clinical Analysis, Santa Lucía General University Hospital, Cartagena-Murcia, Spain; ^3^Faculty of Health Sciences, UCAM Catholic University San Antonio of Murcia, Spain

## Abstract

**Background:**

Anticoagulation with vitamin K antagonists continues to be a challenging task given the difficulty of achieving a correct time in therapeutic range (TTR). The SAMeTT_2_R_2_ score has been proposed to identify patients that will be good responders. In this study we aimed to analyse clinical and genetic factors involved in a correct level of anticoagulation in patients with atrial fibrillation and thereby potentially improve the diagnostic performance of SAMeTT_2_R_2_ score.

**Methods:**

We prospectively included 212 consecutive patients with nonvalvular atrial fibrillation under treatment with acenocoumarol for at least 6 months that were attended in a cardiology outpatient clinic and were categorized as adherent to medication. We carried out a multivariate regression analysis to detect the independent predictive factors of good control. In all patients* VKORC1*,* CYP2C9*⁎*2*,* CYP2C9*⁎*3*, and* MIR133A2* genotyping was performed.

**Results:**

A total of 128 (60.4%) patients presented TTR <70% (average TTR = 63.2). We identified body mass index (OR 0.94, 95%CI 0.89-0.99, p=0.032) and regular vitamin K intake (OR 0.53, 95%CI 0.28-0.99, p= 0.046) as independent predictors of poor anticoagulation control. The discriminatory power of a clinical-genetic model derived from our cohort was significantly better compared to the SAMeTT_2_R_2_ score (C-statistic 0.658 versus 0.524, p<0.001).

**Conclusions:**

In our study the SAMeTT_2_R_2_ score revealed a poor ability in the prediction of TTR. Besides SAMeTT_2_R_2_, body mass index and possibly vitamin K intake should be taken into account when deciding the optimal anticoagulation strategy. The information provided by the identified genotypes was marginal.

## 1. Introduction

The efficacy and safety of treatment with vitamin K antagonists (VKAs) in patients with nonvalvular atrial fibrillation (NVAF) strongly depends on the capability of achieving and maintaining a stable level of correct anticoagulation [1, 2]. Despite the introduction of non-vitamin K antagonist oral anticoagulants (NOAC), the classical VKAs such as acenocoumarol and warfarin remain the most widely used anticoagulant therapy world-wide [3].

Anticoagulation state is monitored by international normalized ratio (INR) whereas quality of anticoagulation is usually assessed with time in therapeutic range (TTR) defined by Rosendaal [4]. It has been proven that the longer the time spent in therapeutic range the lower the risk of emboli or haemorrhage [5]. Although TTR seems to be relatively high in clinical trials, anticoagulation control in the “real-world” is not so strict and TTR results far from optimal [6]. This issue could make NOACs particularly cost-effective when adequate TTR is not achieved with classic anticoagulants [7].

There are very few studies that have appropriately evaluated the quality of anticoagulation in patients with NVAF [8–10]. It is worth noting that assessing quality of anticoagulation is not an easy task since it is a dynamic process with considerable within-patient variation regarding adherence, previous medical status or medical history, pharmacological interactions, and other contributing factors apart from the relatively complex TTR estimation.

Taking all this into consideration, it would be of great interest to have a simple method based on clinical variables that would have the ability to identify those patients under treatment with acenocoumarol that are prone to an inadequate TTR. The SAMeTT_2_R_2_ score has been proposed with such purpose [11]. Authors initially reported that a score of ≥ 2 predicted an inadequate TTR and showed a good discrimination (C-statistic 0.72) in a derivation cohort. However, the SAMeTT_2_R_2_ score was elaborated from a group of patients with AF included in a trial, the AFFIRM study [12], and therefore a potential selection bias (lower age, high proportion of non-Caucasians) is plausible. Moreover, there was a concern due to the well-known “statistical overoptimism” [13]. In an initial attempt to further validate this score, Apostolakis [11] reported a C-statistic of 0.70 (95% CI 0.57-0.82) in a small external cohort. However, a more recent study in a bigger sample [14] has reported lower than expected performance of SAMeTT_2_R_2_ (C-statistic 0.57, 95% CI 0.53-0.60), thus underlying the need to improve the performance of this score.

In this regard, TTR prediction could improve by the determination of genetic polymorphisms involved in the metabolism of acenocoumarol as proved by previous investigations [15]; the most researched ones are* CYP2C9*, (alleles 2 and 3) and rs9923231 (-1639C>T) in* VKORC *[16–19]. Along the same line, the progress in genomic technology and bioinformatics has led to the study of micro-RNA that can play an important role in the response to specific treatments. It has been reported in a small exploratory study that variations in* MIR133A2 *genes can lead to an aberrant expression of* VKORC1, *thus altering the efficacy of treatment with warfarin [20]. Additionally, as mentioned, the seldomly analysed level of patients' adherence as well as other clinical factors might have a role in TTR prediction.

The aim of the present study was to further explore clinical and genetic factors that might be involved in a correct level of anticoagulation with acenocoumarol in patients with NVAF, categorized as adherent according to Morisky-Green scale [21], and thus potentially improve the diagnostic performance of SAMeTT_2_R_2_ score.

## 2. Materials and Methods

### 2.1. Study Population and Definitions

From 1st of December 2014 to 30th of June 2016, we prospectively enrolled consecutive patients diagnosed with NVAF, regardless the type, that were attended at the cardiology outpatient clinic of a tertiary hospital and were under treatment with acenocoumarol for at least 6 months prior inclusion in the study. All patients were considered eligible if they were categorized as “adherent” to the medication according to the Morisky-Green scale (four out of four negative answers in the questionnaire) [21]. Exclusion criteria were (1) moderate or severe rheumatic mitral stenosis, (2) biologic or mechanic mitral valve prothesis, (3) unavailability of INR values for the determination of TTR during the treatment period, (4) recent interruptions (<6 months) of anticoagulation treatment, (5) and anticoagulation with warfarin. The study was performed according to the Declaration of Helsinki and approved by the Ethics Committee for Clinical Research at our centre.

The definitions of other included clinical variables were as follows. Chronic kidney disease was defined as estimated glomerular filtration rate by MDRD-4* <*60 mL/min/1.73 m^2^; chronic hepatic disease was defined as persistent elevation of transaminases 3-fold the upper limit of normal, previous history of cirrhosis, hepatitis, or any other chronic liver disorder. The presence of comorbidities was assessed with the Charlson score [22]. Regular vitamin K intake was defined as consumption of green leafy vegetables such as spinach salad, broccoli, and cabbage, from three to seven days a week. Number of active medications included in patients' standard treatment was quantified as number of tablets taken per day apart from VKA. All abovementioned SAMeTT_2_R_2_ score items were included: female sex, age<60 years, medical history (≥3 comorbidities among the following: hypertension, diabetes mellitus, ischemic heart disease, peripheral arterial disease, heart failure, stroke, pulmonary disease, and liver or renal disease); treatment (interacting drugs such as amiodarone), active smoking, and non-Caucasian race.

### 2.2. Quality of Anticoagulation

INR values of the 6 months prior to the study entry were registered and Rosendaal method [4] was used to assess quality of anticoagulation. Adequate anticoagulation control was defined as an estimated TTR ≥70% [22, 23].

### 2.3. Blood Samples and Laboratory Methods

Peripheral blood samples (5-10 ml) were obtained in EDTA tubes and DNA was extracted using QIAamp DNA minikit and automatic nucleic acid extractor QiaCube (Qiagen, Hilden, Germany). Four SNPs tagging alleles were carefully analysed: rs1799853 (*CYP2C9∗2*), rs1057910 (*CYP2C9∗3*), rs9923231 (-1639C>T* VKORC*), and rs4554 (*MIR133A2*).

Polymorphisms were determined in 96-well plates on a 7500F real-time thermocycler (Applied Biosystems Foster City, CA, USA) using competitive allele-specific PCR (polymerase chain reaction) KASPar probes which are based on FRET (fluorescent resonance energy transfer) technology and following manufacturer's instructions.

## 3. Statistical Analysis

Continuous variables were presented as means (with standard deviations) or medians (with 25th and 75th percentiles). Categorical variables were expressed as frequencies and percentages. The Kolmogorov-Smirnov test and frequency histograms were applied to establish the normality of the included variables. Baseline characteristics were compared between patients with adequate (TTR≥70%) or inadequate (TTR<70%) anticoagulation control. Continuous variables were compared with Student's t test or Mann-Whitney test as appropriate. Categorical variables were compared with Chi-square or exact Fisher test. We used a binary logistic regression model to predict a TTR<70% (dependent variable) including both clinical and genetic variables. Assumptions of the model were previously tested. Odds ratio and 95% confidence interval (CI) were calculated for each covariate as well as the discrimination of the model by using C-statistic (estimation of the area under the curve (AUC)) and the Hosmer-Lemeshow test to assess calibration. We chose variables with p<0.15 to develop a multivariate regression model using* backward method *for the clinical variables and* enter method *for the genetic variables. Likelihood ratio test was used to assess the significance of each variable. To further check the consistency of the multivariable model we performed a 3000-iteration bootstrapped enter method analysis. In all tests, a two-sided p-value 0.05 was considered significant. Software packages SPSS 21.0 (SPSS Inc., Chicago, IL, USA) and STATA 12.0 (StataCorp, USA) were used for the statistical analyses.

## 4. Results

### 4.1. Baseline Characteristics

We included two hundred and twelve patients with a mean age of 74 years (standard deviation of 9 years), and 105 (50%) were men. Baseline characteristics of the study population are shown in Table 1. A total of 128 (60.4%, 95% CI 53.7-67.0%) patients presented TTR <70%. The mean TTR was 63.2 (standard deviation 20.3). Mean SAMeTT_2_R_2_ score was 1.3 ± 1.0 (median 1, p25-p75 1-2, range 4).

### 4.2. Predictors of Poor Anticoagulation Control

Body mass index (OR 0.93, 95%CI 0.88-0.99, p=0.015), previous heart failure (OR 1.94, 95%CI 0.91-4.15 p= 0.085), regular vitamin K intake (OR 0.52, 95%CI 0.28-0.95, p=0.032), persistent or permanent AF (OR 1.74, 95%CI 0.99-3.05, p=0.055), and number of active medications (OR 1.09, 95%CI 0.99-1.20, p=0.090) were associated (p<0.15) with a TTR <70% (Table 3). In a multivariable setting we found that body mass index (OR 0.94, 95%CI 0.89-0.99, p=0.032) and regular vitamin K intake (OR 0.53, 95%CI 0.28-0.99, p=0.046) were independent predictors of poor anticoagulation control in a correctly calibrated multivariable model (Table 4). In the bootstrapped model, only body mass index remained as an independent predictor (OR 0.94, 95% CI 0.87-0.99, p=0.044).

### 4.3. Polymorphisms. Frequency and Impact on TTR

The genotype distribution of the* VKORC *polymorphism was CC 35%, CT 44% and TT 21%.* CYP2C9∗2 *presented genotype frequencies of CC 65%, CT 33%, TT 2%. Genotype frequency of* CYP2C9∗3 *was AA 84% and CA 16%. Finally,* MIR133A2 *genotype was distributed as follows, GG 60%, AA 11%, GA 28%, undetermined 1%.

The frequencies of* VKORC, CYP2C9∗2, CYP2C9∗3, *and* MIR133A2 *polymorphisms according to TTR are shown in Table 2. Patients with poor anticoagulation control presented a trend towards a higher prevalence of at least one T allele in the* VKORC *polymorphism. Notably, all genotype frequencies agreed with the Hardy-Weinberg equilibrium p=0.57 (*X*^2^ =2.38), p=0.81 (*X*^2^ =1.31), p=0.92 (*X*^2^ =1.71), and p=0.75 (*X*^2^=14.89), respectively.

### 4.4. Diagnostic Performance of SAMeTT_2_R_2_ Score and a Clinical-Genetic Model

**Figure 1:**
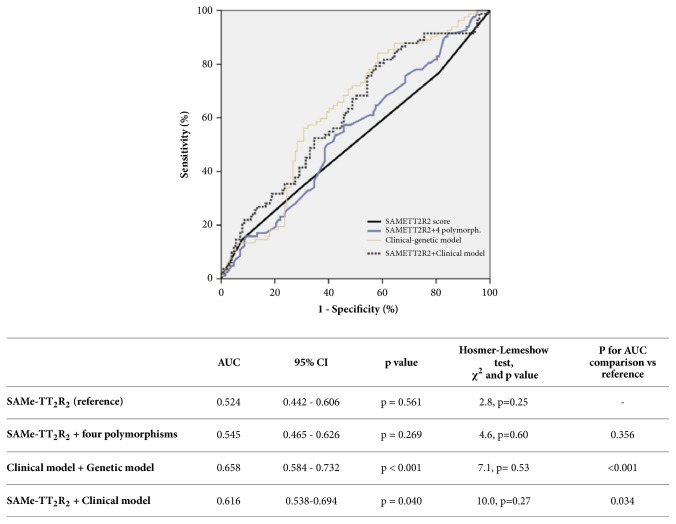
Diagnostic performance of SAMe-TT_2_R_2_ and SAMe-TT_2_R_2_ plus four polymorphisms/clinical model and clinical-genetic model in the detection of TTR≥70%. AUC, area under the curve; CI, confidence interval. The four included polymorphisms are* VKORC, CYP2C9∗2, CYP2C9∗*3, and* MIR133A2*. The clinical model comprises body mass index (kg/m^2^) and regular vitamin K intake.

Discrimination of SAMeTT_2_R_2_ as reflected by the C-statistic demonstrated a poor performance in our study population (AUC 0.524 95% CI 0.442-0.606) to detect a TTR≥70%. Moreover, C- statistic for the model that included SAMeTT_2_R_2_ score plus four analysed polymorphisms was 0.545 (95% CI 0.465-0.626; p=0.269 for comparison with SAMeTT_2_R_2_). Finally, the AUC corresponding to the model that included two clinical variables (body mass index and regular vitamin K intake) and four polymorphisms was 0.658 (95%CI 0.584-0.732). The increment of the discrimination capacity yielded by the clinical-genetic model compared to SAMeTT_2_R_2_ score alone was 28.8%, p<0.001. Also, the discrimination capacity of the clinical model and the SAMeTT_2_R_2_ score above the SAMeTT_2_R_2_ alone was 20.5% (p=0.034) (Figure 1).

## 5. Discussion

The first and still the most widely used anticoagulants are the classical VKAs such as acenocoumarol and warfarin [24]. Thus, we believe that it seems reasonable to continue exploring the quality of anticoagulation and the possible factors involved in a poor control of such therapy. In this regard Apostolakis et al. presented the aforementioned SAMeTT_2_R_2_ score [11]. Apart from the derivation and internal validation cohorts, the author analysed the SAMeTT_2_R_2_ score performance in a smaller external “real-world” validation cohort. The C-statistic of 0.70 (95%CI 0.57-0.82) reported for this cohort was calculated for discrimination of the 5th percentile (TTR≥64%) of this sample. This notorious result was in contrast with the large nationwide study conducted by Ruiz- Ortiz et al. [14] with a C-statistic of 0.57(95%CI 0.53-0.60) for the prediction of TTR≥65%. Consistently, in our study, SAMeTT_2_R_2_ showed a C-statistic of 0.549 (95% CI 0.472-0.626) for the prediction of TTR ≥ 65%. Notably, our results are in consonance with the poor predictive ability of SAMeTT_2_R_2_ score reported in a high-quality setting of a Danish cohort by Jane Skovet al. [25] who applied SAMeTT_2_R_2_ score to a small cohort of patients with a mean TTR of 76%. SAMeTT_2_R_2_ score showed a very low prediction of TTR (adjusted R^2^= 4%) while the use of a model that included age, amiodarone use, alcohol consumption, and perceived stress showed more than double the R^2^ value. Anticoagulation therapy is not exempt from complications as bleeding and drawbacks such as the need of monitoring and interactions with vitamin-K-rich food, aspects that may affect adherence to such therapy. The impact of the adherence to anticoagulants in the performance of the prediction tools for TTR has not been sufficiently analysed. Thus, in an attempt to overcome this pitfall, we used Morisky-Green scale so as to include only “adherent” patients. It has been reported that this scale shows low sensitivity but high specificity and positive predictive value [26]. To the best of our knowledge, these types of medication adherence scales have not been previously used in other studies in a setting similar to ours.

Clearly, decision making in anticoagulation therapy cannot be left to low performance scores, so the search for more precise predictors in guaranteed. In this regard, we found that the combination of only two clinical variables (body mass index and regular vitamin K intake) and four genetic polymorphisms modestly—but significantly—improved diagnostic performance of SAMeTT_2_R_2_ score (C-statistic = 0.658, 95% CI 0.584-0.732). Along the same line, Abumuaileq et al. carried out a retrospective analysis of a real-world cohort of patients with NVAF [27]. The SAMeTT_2_R_2_ score C-statistic was evidently poor and mildly improved from 0.56 to 0.60 by adding new factors such as alcohol abuse, low glomerular filtration rate, diabetes mellitus, heart failure, and history of malignancy in accordance with previous reports [28]. However, the study had limitations since authors used the percentage of INR in therapeutic range (PINRR) not completely equivalent to TTR.

Another study worth mentioning is the one published by Lobos-Bejarano et al. [29] with a large sample size based on the PAULA cohort [30]. By applying SAMeTT_2_R_2_ score in a real-life scenario they confirm its modest prediction capability of INR control (C-index 0.54-0.58) and they identify easy-to-collect factors (seven or more tablets per day, dietary habits, and bleeding history) capable of improving it vaguely. The implication of dietary habits that include vitamin K-rich foods (OR 0.52, 95% CI 0.28-0.95) and number of active medications (OR 1.09, 95% CI 0.99-1.20) are two factors that also have shown to be involved in TTR control in our study.

An interesting finding was recently reported by Bryk et al. [31] and is substantial in countries like Spain where the most widely used VKA is acenocoumarol. By comparing the predictive ability of SAMeTT_2_R_2_ score in patients with AF treated with warfarin versus acenocoumarol, they detect that it is less effective in predicting unstable anticoagulation with the latter and improves significantly by adding statin use and the presence of COPD (0.66; 95% CI 0.58-0.73 versus 0.56; 0.48-0.64, p = 0.042). This finding could explain at least partially the worse performance of SAMeTT_2_R_2_ score in countries that use acenocoumarol in opposition to those using warfarin like the population included in the AFFIRM study.

The present study shows that 62.5% of our patients presented TTR <70%. Various study groups over the years have tried to take up the challenge of analysing quality of anticoagulation with varying results. Among Spanish groups, FANTASIIA [8], CALIFA [14], PAULA [30], and ANFGAL [32] studies are retrospective registries that claim that only around 50% of patients achieve a correct TTR. However, the identified factors involved in poor anticoagulation control are diverse and not always consistent.

We identified regular vitamin K intake and BMI as predictors of poor anticoagulation control, being the latter the most consistent as shown by the bootstrapped analysis. During the last several years, multiple studies have attempted to detect the most important clinical, demographic, and even genetic factors capable of predicting anticoagulation control. The abovementioned factors identified in our study are in line with previous findings. In a post hoc analysis of the AMADEUS trial Senoo et al. [33] describes the importance of BMI on the quality of anticoagulation where paradoxically obesity was associated with better anticoagulation control. The reason of such association remains elusive. It has also been suggested that until the completion of further studies, obese patients should be treated with VKA as first option given the weight dependent modifications in drug clearance and the possibility of subtherapeutic drug levels while using NOACs [34].

Regarding the interaction between VKA and vitamin K-rich foods, a systematic review recently published by Violi et al. [35] sustains that the prevailing advice to modify dietary habits in patients with VKAs is not sufficiently supported by up-to-date evidence. In line with other studies [36, 37] they suggest the maintenance of a stable dietary habit or even vitamin K supplementation in order to avoid major changes in the intake of vitamin K. Certainly, vitamin K has already been reported to be an independent predictor of high-quality oral anticoagulation by other authors [38].

Authors have claimed that the clinical prediction tools that are at hand can only explain less than 10% of the variability behind poor anticoagulation control [6], hence genetic variations might play a role in INR stability. Such is the importance of genetic predisposition, that the European Medicines Agency gathers the effect of certain* VKORC *and* CYP2C9 *variants on the pharmacokinetics of warfarin and suggests that genotype information may assist dose selection and reduce time to reach target INR [39]. Likewise, the Food and Drug Administration released a communication advising clinicians to consider genetic testing prior initiation of warfarin therapy [40]. Nevertheless, the cost-effectiveness of this approach is debatable. In addition, two validated dosing algorithms (i.e., Gage and IWPC) have been proposed in an updated guideline by the Clinical Pharmacogenetics Implementation Consortium [41].

In this line a very recent meta-analysis of genotype-guided versus standard dosing of VKA [15] reveals that indeed the former can improve TTR in addition to the reduction of risk for bleeding events. However, we must bear in mind that the inclusion of patients that received anticoagulant treatment for at least 6 months might have limited the potential value of genotyping that in fact might be more significant at the start of the therapy. Further, the Chinese group of Liu et al. sought to improve SAMeTT_2_R_2_ score by replacing “the non-white race” variable with the most representative genotypes, ascending the C-index from 0.60 to 0.67. Therewith, the authors suggest that the modified SAMeTT_2_R_2_ score could be more appropriate in a racial diverse group of patients [42].

We expanded our genetic analysis to rs4554* MIR133A2 *genotyping; unfortunately, we did not find an association between this polymorphism and TTR. In spite of a similar number of analysed patients, the aforementioned exploratory study that examined the effect of variations in* MIR133A2 *[20] was conducted in a different population to ours (e.g., younger age, absence of concomitant drugs that could alter INR values). In addition, we must take into account the fact that the concept of “one miRNA regulating one gene modifying the efficacy of one drug” might not consider the full extent of miRNA regulation. Nevertheless, future studies in larger samples are warranted. Hence a thorough elaboration of optimized clinical models along with the comprehension of the implication of genetic polymorphisms could play a role for the future of personalized medicine in the field of anticoagulation.

## 6. Strengths and Limitations

Several limitations of the present study must be noted: first, those regarding the study design such as retrospective character and single centre that limited the evaluation of less common predictors. Another peculiarity of our study is that the predominant VKA in Spain is acenocoumarol, as opposed to other countries, where warfarin is the VKA of choice. Subtle differences in their pharmacokinetics and pharmacodynamics hamper the use of the exact same dosing algorithms. However, our study had strengths. We included a cohort of consecutive patients that were considered adherent to the medication, potentially reducing a risk of bias in this regard. Also, we performed a careful evaluation of previous medical background and explored the usefulness of the* MIR133A2 *polymorphism, over and above that of* VKORC1, CYP2C9∗2*, and* CYP2C9∗3*.

## 7. Conclusions

In this study, SAMeTT_2_R_2_ score showed a poor diagnostic performance in the prediction of TTR. We identified body mass index and regular vitamin K intake as factors that could improve SAMeTT_2_R_2_ score. Finally, although the information provided by the identified genotypes is marginal in our study, the progressive availability of genetic testing could become a promising tool for the future.

## Figures and Tables

**Table 1:** Baseline Characteristics according to time in therapeutic range.

	**Total cohort (n=212)**	**TTR <70**%** (n=128, 60.4**%**)**	**TTR ≥70**%** (n=84, 39.6**%**)**	**p-value**
**Age, years**	74 ± 9.0	74 ± 8.8	73 ± 9.2	0.286
**Male gender, n (**%**)**	105 (50.0)	63 (49.2)	42 (50.0)	0.911
**Body mass index, kg/m** ^**2**^	30.9 ± 5.2	30.2 ± 4.8	32 ± 5.5	0.013
**Current smoking, n (**%**)**	22 (10.4)	14 (10.9)	8 (9.5)	0.741
**Dyslipidaemia, n (**%**)**	100 (47.2)	58 (45.3)	42 (50.0)	0.504
**Hypertension, n (**%**)**	158 (75.0)	91 (71.1)	67 (79.8)	0.157
**Type 2 diabetes mellitus, n (**%**)**	63 (29.7)	34 (26.6)	29 (34.5)	0.215
**Previous heart failure, n (**%**)**	40 (18.9)	29 (22.7)	11 (13.1)	0.082
**Previous stroke, n (**%**)**	20 (9.4)	14 (10.9)	6 (7.1)	0.355
**Previous ischemic heart disease, n (**%**)**	47 (22.2)	29 (22.7)	18 (21.4)	0.833
**Previous peripheral arterial disease, n (**%**)**	16 (7.5)	11 (8.6)	5 (6.0)	0.476
**Previous pulmonary disease, n (**%**)**	51 (24.1)	33 (25.8)	18 (21.4)	0.468
**Previous renal disease, n (**%**)**	27 (12.7)	18 (14.1)	9 (10.7)	0.474
**Previous hepatic disease, n (**%**)**	1 (0.5)	1 (0.8)	0	1
**Previous neoplasm, n (**%**)**	22 (10.4)	11(8.6)	11 (13.1)	0.293
**Charlson score**	1.3 ± 1.5	1.4 ± 1.5	1.3 ± 1.4	0.505
**Alcohol intake, grams per day**	4.2 ± 7.1	4.2 ± 7.5	4.2 ± 6.5	0.951
**Regular vitamin K intake**	138 (65.1)	76 (59.4)	62 (73.8)	0.031
**Persistent or permanent atrial fibrillation, n (**%**)**	128 (60.4)	84 (65.6)	44 (52.4)	0.054
**SAMe-TT**_**2**_ **R**_**2**_ ** score**	1.3 ± 1.0	1.2 ±0.9	1.3 ± 1.1	0.522
**Age < 60 years, n (**%**)**	15 (7.1)	7 (5.5)	8 (9.5)	0.260
**Medical history, n (**%**)**	64 (30.2)	38 (29.7)	26 (31.0)	0.844
**Treatment (interacting medications), n (**%**)**	58 (27.4)	35 (27.3)	23 (27.4)	0.995
**Non-Caucasian race, n (**%**)**	1 (0.5)	1 (0.8)	0	1
**Number of active medications**	7.3 ± 3.0	7.6 ± 3.1	6.9 ± 2.9	0.088
**≥ 7 active medications** **∗** ** (**%**)**	112 (52.8)	72 (56.3)	40 (47.6)	0.218
**Estimated glomerular filtration rate, ml/min/1.73m** ^**2**^	73.6 ± 26.4	72.6 ± 24.6	75.2 ±29.1	0.487

TTR, time in therapeutic range; CI, confidence interval. Medical history: hypertension, diabetes mellitus, ischemic heart disease, peripheral arterial disease, heart failure, previous stroke, pulmonary disease, and liver or renal disease. Number of active medications: number of tablets taken per day apart from VKA. *∗*7 corresponds to the median value of number of active medications.

**Table 2:** Proportion of polymorphisms according to time in therapeutic range.

	**Total cohort (n=212)**	**TTR <70**%** (n=128, 60.4**%**)**	**TTR ≥70**%** (n=84, 39.6**%**)**	**p-value**
***VKORC *≥ 1 allele T, (TT/CT), n (**%**)**	138 (65.1)	85 (66.4)	53 (63.1)	0.621
***CYP2C9*** **∗** ***2 *≥1 allele T (TT/CT), n (**%**)**	75 (35.4)	44 (34.4)	31 (36.9)	0.706
***CYP2C9*** **∗** ***3 *≥ 1 allele C (CC/CA), n (**%**)**	35 (16.5)	20 (15.6)	15 (17.9)	0.669
***MIR133A2 ≥ *1 allele A (AA/GG), n (**%**)**	82 (38.7)	51 (39.8)	31 (36.9)	0.667

TTR, time in therapeutic range.

**Table 3:** Unadjusted logistic regression model: predictors of time in therapeutic range <70%.

	**Odds Ratio**	**95**%** CI**	**p-value**
**Clinical variables**			
**Age, years**	1.02	0.99 - 1.05	0.285
**Male gender, n (**%**)**	1.03	0.60 - 1.79	0.911
**Body mass index, kg/m** ^**2**^	0.93	0.88 - 0.99	0.015
**Current smoking, n (**%**)**	1.17	0.47 - 2.92	0.741
**Dyslipidaemia, n (**%**)**	0.83	0.48 - 1.44	0.504
**Hypertension, n (**%**)**	0.62	0.32 - 1.20	0.158
**Type 2 diabetes mellitus, n (**%**)**	0.69	0.38 - 1.25	0.216
**Previous heart failure, n (**%**)**	1.94	0.91 - 4.15	0.085
**Previous stroke, n (**%**)**	1.60	0.59 - 4.33	0.359
**Previous ischemic heart disease, n (**%**)**	1.07	0.55 - 2.09	0.833
**Previous peripheral arterial disease, n (**%**)**	1.49	0.50 - 4.44	0.479
**Previous pulmonary disease, n (**%**)**	1.27	0.66 - 2.45	0.469
**Previous renal disease, n (**%**)**	1.36	0.58 - 3.20	0.476
**Previous hepatic disease, n (**%**)**	-	-	-
**Previous neoplasm, n (**%**)**	0.62	0.26 - 1.51	0.296
**Charlson score**	1.07	0.88 - 1.30	0.504
**Alcohol intake, grams per day**	1.00	0.96 - 1.04	0.952
**Regular vitamin K intake**	0.52	0.28 - 0.95	0.032
**Persistent or permanent atrial fibrillation, n (**%**)**	1.74	0.99 - 3.05	0.055
**SAMe-TT**_**2**_ **R**_**2**_ ** score**	0.91	0.68 - 1.21	0.504
**Age < 60 years, n (**%**)**	0.55	0.19 - 1.58	0.266
**Medical history, n (**%**)**	0.94	0.52 – 1.71	0.844
**Treatment (interacting medications), n (**%**)**	1.00	0.54 - 1.85	0.995
**Non-Caucasian race, n (**%**)**	-	-	-
**Number of active medications**	1.09	0.99 - 1.20	0.090
**≥ 7 active medications** **∗** ** (**%**)**	1.41	0.81 - 2.46	0.219
**Estimated glomerular filtration rate, ml/min/1.73m** ^**2**^	1.00	0.99 - 1.01	0.487
**Genetic variables**			
***VKORC *≥ 1 allele T (n=212)**	1.16	0.65 - 2.06	0.621
***CYP2C9*** **∗** ***2 *≥ 1 allele T (n=212)**	0.90	0.50 - 1.59	0.706
***CYP2C9*** **∗** ***3 *≥ 1 allele C (n=212)**	0.85	0.41 - 1.78	0.669
***MIR133A2 *≥ 1 allele A (n=212)**	1.13	0.64 - 2.00	0.667

CI, confidence interval. Medical history: hypertension, diabetes mellitus, ischemic heart disease, peripheral arterial disease, heart failure, previous stroke, pulmonary disease, and liver or renal disease. Number of active medications: number of tablets taken per day apart from VKA. *∗*7 corresponds to the median value of number of active medications.

**Table 4:** Multivariate logistic regression model for the prediction of time in therapeutic range <70%.

	**Odds Ratio ** **∗**	**95**%** CI**	**P value**
**Clinical variables**			
** Body mass index, kg/m** ^**2**^	0.94	0.89 - 0.99	0.032
** Regular vitamin K intake**	0.53	0.28 - 0.99	0.046
**Genetic variables**			
** *VKORC *≥ 1 allele T (n=212)**	1.18	0.65 - 2.16	0.591
** *CYP2C9*** **∗** ***2 *≥ 1 allele T (n=212)**	1.10	0.59 - 2.02	0.772
** *CYP2C9*** **∗** ***3 *≥ 1 allele C (n=212)**	0.80	0.37 - 1.72	0.568
** *MIR133A2 *≥ 1 allele A (n=212)**	1.11	0.61 - 2.03	0.724

*∗*Adjusted by previous heart failure and number of active medications. Hosmer-Lemeshow: *χ*^2^ = 7.072, p= 0.529.

## Data Availability

The data that support the findings of this study are available from the corresponding author [Luciano Consuegra-Sánchez] upon reasonable request.

## Abbreviations and Acronyms

AF: Atrial fibrillation
AUC: Area under the curve
CI: Confidence interval
COPD: Chronic obstructive pulmonary disease
INR: International normalized ratio
mRNA: Microribonucleic acid
NOAC: Non-vitamin K antagonist oral anticoagulant
NVAF: Nonvalvular atrial fibrillation
TTR: Time in therapeutic range
VKAs: Vitamin K antagonists.
